# Polymer physics of chromosome large-scale 3D organisation

**DOI:** 10.1038/srep29775

**Published:** 2016-07-13

**Authors:** Andrea M. Chiariello, Carlo Annunziatella, Simona Bianco, Andrea Esposito, Mario Nicodemi

**Affiliations:** 1 Dipartimento di Fisica, Università di Napoli Federico II, and INFN Napoli, CNR-SPIN, Complesso Universitario di Monte Sant’Angelo, 80126 Naples, Italy

## Abstract

Chromosomes have a complex architecture in the cell nucleus, which serves vital functional purposes, yet its structure and folding mechanisms remain still incompletely understood. Here we show that genome-wide chromatin architecture data, as mapped by Hi-C methods across mammalian cell types and chromosomes, are well described by classical scaling concepts of polymer physics, from the sub-Mb to chromosomal scales. Chromatin is a complex mixture of different regions, folded in the conformational classes predicted by polymer thermodynamics. The contact matrix of the *Sox9* locus, a region linked to severe human congenital diseases, is derived with high accuracy in mESCs and its molecular determinants identified by the theory; *Sox9* self-assembles hierarchically in higher-order domains, involving abundant many-body contacts. Our approach is also applied to the *Bmp7* locus. Finally, the model predictions on the effects of mutations on folding are tested against available data on a deletion in the *Xist* locus. Our results can help progressing new diagnostic tools for diseases linked to chromatin misfolding.

New technologies, such as Hi-C methods, are revealing that the genome of mammalian cells has a complex 3D architecture, comprising extensive long-range, functional interactions^1^,^2^,^3^,^4^,^5^. Chromosomes are folded into a sequence of 0.5–1.0 Mb long domains, known as TADs^6^,^7^, which are comparatively conserved between mice and humans. TADs, in turn, are thought to be one level of a hierarchy of higher-order domains (metaTADs), extending up to chromosomal scales^8^,^9^,^10^. Chromatin interactions have key functional roles, as for instance they control gene activity through the formation of physical loops between regulatory regions and target remote genes, disruptions being linked to human conditions^11^,^12^. Yet, the basic molecular mechanisms whereby chromosome 3D organisation is orchestrated in health and disease remain mysterious.

To make sense of genome-wide contact data and to expose the principles shaping chromosome 3D structure, models from polymer physics have been introduced^13^. Steric hindrance effects have been shown to play important roles in chromatin folding^2^,^14^,^15^,^16^, yet interacting polymer models have to be considered to describe FISH and Hi-C data in a more quantitative way^17^,^18^,^19^,^20^,^21^,^22^,^23^. In particular, we here we focus on the *Strings&Binders* (SBS) model of chromosome folding^18^ (Fig. 1a), a polymer model where the formation of chromatin loops is determined by specific interactions with DNA-binding molecules. The SBS model was the first to recapitulate within a single framework Hi-C and FISH data^10^,^13^,^20^.

Here we show that scaling concepts of classical polymer physics can explain the large scale behaviour of contact data over three orders of magnitudes in genomic separation, across different cell types and chromosomes. Chromatin is a complex mixture of different regions, folded in classes as predicted by polymer physics. The contact matrices of the *Sox9* locus, a region associated to severe congenital diseases^11^, and of the *Bmp7* locus, important in tissue development, are derived with high accuracy in mESC. Our models informed with genomic rearrangements can be used to anticipate their effects on the 3D architecture from only polymer physics. We test our *in-silico* predictions in the case of the Δ*XTX* deletion in the *Xist* locus where 5C data are available^7^. As our theory identifies the molecular determinants of folding, it can help progressing new diagnostic tools for diseases linked to chromatin misfolding, such as cancer and congenital disorders.

## Results

**Figure 1:**
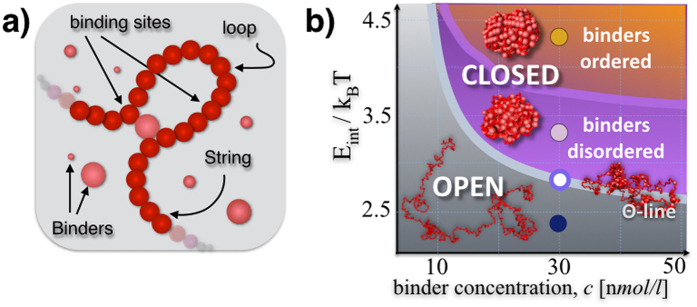
A polymer model of chromatin. (**a**) The Strings&Binders (SBS) model is a Self-Avoiding (SAW) chain of beads interacting with molecular binders having a concentration, *c*, and a binding affinity *E*_*Int*_. (**b**) Polymer physics dictates that the model different stable architectural classes correspond to the different phases of its phase diagram: at low *E*_*Int*_ or *c*, the polymer is open and randomly folded in its *coil* phase; above its Θ-point transition, in the *globule* phase it is closed in more compact conformations as signalled by a drop in its gyration radius (Figure S1a); in the closed state, at higher values of *E*_*Int*_ or *c*, its binders have a transition form a disordered to an ordered arrangement, as shown by their Structure Factor (Figure S1b).

In the SBS model, a chromatin filament is represented at a coarse-grained level as a Self-Avoiding (SAW) string of beads interacting with diffusing binders (Fig. 1a). We use the interaction potentials developed in classical studies of polymer physics^24^. The beads of the chain, each having a diameter σ, are connected by FENE bonds^24^ (see Supplementary Materials and Methods). The beads interact with the diffusing molecular binders through a Lennard-Jones attractive potential, having an energy scale, *E*_*int*_. The binders have a concentration, *c* (and, for simplicity, have the same diameter σ); they can bridge the beads of the chain and, thus, fold the polymer. Each chromosome bead and each binder is subject to Brownian motion, here investigated by Molecular Dynamics (MD) computer simulations via LAMMPS^25^ in a box with periodic boundary conditions. To model a coarse-grained chromosome, we initially consider chains of N = 1000 beads; so, for instance, a 100 Mb long chromosome would have beads σ = 87 nm wide, each encompassing 100 k bases. In all cases, we work at scales much larger than the chromatin persistence length, where polymer scaling concepts^26^ are expected to apply.

### Phase diagram and conformational classes

To characterize the thermodynamics features of the model, we first focus on the simple case of a homopolymer with identical beads, interacting with one type of DNA binders. In the space of the control parameters, *E*_*int*_ and *c*, the polymer has a known coil-globule folding transition^13^,^26^ signaled by a sharp drop in the value of its gyration radius at the Θ-point (Supplementary Figure S1a, and Supplementary Materials and Methods). In the coil state, observed at small *E*_*int*_ and *c*, the binders do not succeed in establishing stable loops and the polymer has an open conformation falling in the universality class of the SAW (Fig. 1b). In the globular state, conversely, the binders fold the polymer in a closed conformation, occupying approximately 1% of the open state volume.

Here we discuss a new phase-transition, occurring in the polymer globular phase, wherby the binders, albeit not interacting directly with each other, undergo an order-disorder transition, which affect the finer structure of folding. At least two states exist: at low interaction energies or concentrations, the binders form a disordered lump around the chain, with a homogeneous density distribution and a flat structure function, *S*(*k*); at higher *E*_*int*_ or *c*, they form instead an ordered aggregate, revealed by sharp peaks in *S*(*k*) (Figure S1b, and Materials and Methods). The phase diagram of the polymer-binders system is summarized in Fig. 1b: as dictated by polymer physics, the thermodynamics phases define the system stable conformational classes, which are expected to play an important role in the way chromatin folds. The phase transitions energies and corresponding concentrations fall in the weak biochemical energy range and in the nano- to micromole/litre range, as expected from biological considerations (Fig. 1b).

To characterize the folding state of our polymer model, we computed the average pairwise contact probability, *P*_*c*_(*s*), of bead pairs at a given contour distance, *s*. *P*_*c*_(*s*) only depends on the thermodynamics state of the system (Figure S2). In the coil state, *P*_*c*_(*s*) decreases asymptotically as a power law with *s*, *P*_*c*_(*s*) *~* *s*^*−α*^, with an exponent *α* *~* 2.1 in the SAW universality class. At the Θ-point, the exponent becomes *α* ~ 1.5, as known in polymer physics. In the globule state, *P*_*c*_(*s*) depends on whether the system is in the disordered state, where after an initial decrease, a long plateau is found, or whether it is in the ordered state, where an exponent close to *α* ~ 1.0 is observed. Analogously, the mean square distance of site pairs, *R*^*2*^(*s*), which can be accessed by FISH measurements, depends on the system thermodynamics phase (Figure S3, Supplementary Materials and Methods).

The coil-globule and order-disorder transitions are general features of the physics of interacting polymers, and more specific thermodynamics phases are known to exist depending on the molecular specificities of the system considered^26^. Even within our simplified context, the details of polymer conformations depend on other aspects, including the positioning of the binding sites along the bead chain, which could be unequally spaced and interspersed by ‘inert’ (non-interacting) sites, or local crowding and confinement. Different distributions of binding sites would produce, for instance, different types of ‘rosette-like’ globular conformations, with varying ranges of loop lengths. Furthermore, off-equilibrium, unstable conformations are also expected to be encountered in real chromosomal regions, in particular during changes in the folding state.

### Chromatin is a mixture of different regions folded in their thermodynamics states

**Figure 2:**
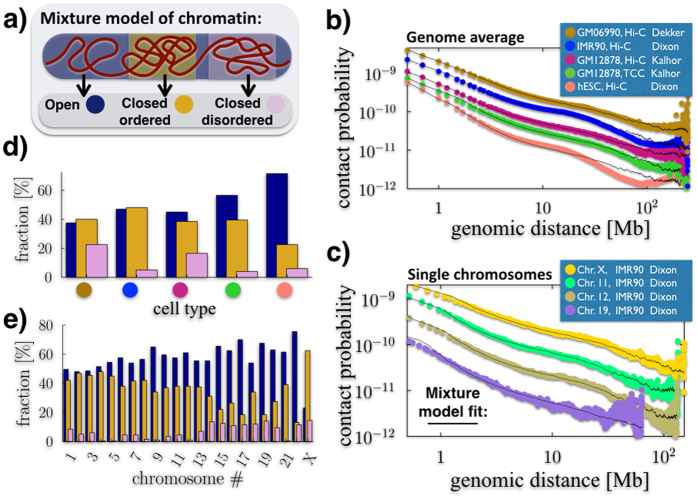
Chromatin is a mixture of regions folded in different thermodynamics states. (**a**) We model a chromatin filament as a mixture of differently folded regions, each belonging to one of the stable conformational classes envisaged by polymer physics (Fig. 1). In this view, the average pairwise contact probability is only determined by the relative abundances of the states in the mixture, as each state has a fixed, specific pairwise contact probability (Figure S2). (**b**) Genome-wide average contact frequencies across human cell types, from Hi-C and TCC technologies, can be fitted from the sub-Mb to chromosomal scales by such a mixture model. (**c**) Single chromosome data (here from IMR90 cells) can be similarly explained. (**d**) Different cell types (see colour scheme in panel b) have a different chromatin composition, with hESC (orange circle) more open than differentiated cells, such as IMR90 (blue circle). (**e**) Within a given cell type (here IMR90, as in **c**) distinct chromosomes have also a different composition, with chromosome X formed mostly of closed regions, whereas gene rich chromosomes, e.g., chr.19, are up to 70% open.

To compare our model against Hi-C data, we reasoned that a single chromosome is likely to be a mixture of a variety of different folded regions, including for instance eu- and heterochromatin domains, which can dynamically change from cell to cell according to functional purposes^27^. Yet, the stable spatial conformations of such regions must belong to one of folding classes determined by polymer physics (pure states), at least as a first approximation. To model such a scenario, we considered a mixture polymer system composed of different chain segments, each folded in one of the given thermodynamics states identified above (Fig. 2a).

To test the biological significance of such a model of chromatin, we compared its predicted pairwise contact probability, *P*(*s*), with available Hi-C, TCC and *in-situ* Hi-C contact frequency data. In our mixture model, *P*(*s*) is just a linear combination of the contact probabilities of the pure states, independently derived above. It only depends on the relative abundances of the states in the mixture (and on a scale factor used to map bead sizes into genomic separations, see Supplementary Materials and Methods). We find that such a model can fit genome-wide averaged data (Fig. 2b, χ^2^ < 8 × 10^−3^) and single chromosome data (Fig. 2c, χ^2^ < 1.4 × 10^−1^) over approximately three orders of magnitude in length, from 0.5 Mb to chromosomal scales, across a variety of different cell types and experimental techniques.

Our approach also returns the mixture composition that best describes the given data (Fig. 2d,e, error bars are below 10% of signal, not shown for clarity). We find that different cell types have varying fractions of open state chromatin: embryonic stem cells^6^ (hESC) have the highest one, around 75%, while differentiated cells (e.g., IMR90) have values closer to 50%, in agreement with expectations. Different techniques (Hi-C v.s. TCC) give overall similar results in the same cell type: for instance, in GM12878 cells the fraction of closed ordered chromatin is 40% in both Hi-C and TCC data, yet the other states have a slightly different balance in the two cases. Different chromosomes can have very different compositions: in IMR90 cells, for instance, chromosome X is typically very compact (75%) with a prevalence of the closed states. In general, the shorter the chromosome the higher is its open fraction. For example, chromosome 1 is only 50% open; chromosome 11 or 12 are 40% in the closed-ordered conformation, with less than 5% in the disordered state; the gene rich chromosome 19 is one of the less compact (70% open), with the ordered and disordered closed states present in a 3/2 ratio. In brief, the mixture composition reflects the distribution of different folding domains along the chromosomes in the different cell types, across their thermodynamics states. Later, we will discuss the nature of the specific folding domains of important genomic loci, such as *Sox9*, *Bmp7* and *Xist*.

### Self-assembly of topological domains and higher-order structures

**Figure 3:**
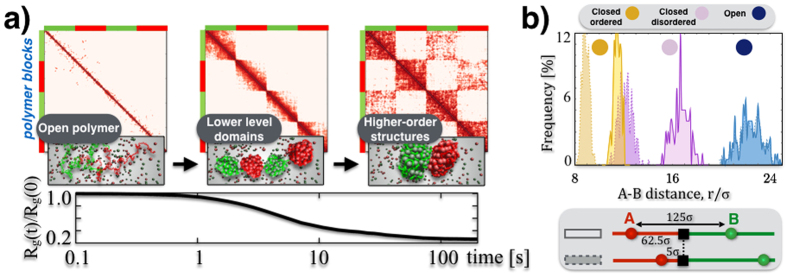
Higher-order domains and symmetry breaking architectures. (**a**) We consider a block-copolymer composed of red and green binding domains which spontaneously folds, from an initially SAW conformation, in the ordered closed state of Fig. 1 (here *c* = 54 n*mol/l* and *E*_*int*_ = 4.1 k_B_T). The process is marked by a decrease of the gyration radius, *R*_*g*_(*t*), in time (bottom panel) and by the formation of a hierarchy of higher-order domains, as reflected by the contact matrix pattern (top). (**b**) Pairs of sites with the same contour separation, differently positioned across a block boundary (see bottom panel), have the same average physical distances, r, in the open phase. Yet, in the closed states, the symmetry is broken by their different position relative to the boundary as the two pairs have a different physical distance, as seen from the corresponding distributions of r (from left to right *E*_*int*_ = 4.1, 3.1, 0 k_B_T, and *c* = 54 n*mol/l*).

To investigate the patterns of Hi-C matrices beyond the average contact probability, we next studied the mechanisms underlying the self-assembly of topological domains. The average pairwise contact matrix of our homopolymer is different in the different thermodynamics phases and, as expected, the contact network is strongly enhanced in the closed states (Figure S4). We next considered the case of a block-copolymer with two types of beads (red/green) alternated in two pairs of blocks along the polymer chain, interacting each with a different type of binders (red/green, Fig. 3a, see Supplementary Materials and Methods). Each polymer block can fold in the conformational states discussed for the homopolymer. The random encounter of beads within the same block is favored by genomic proximity, resulting, above the Θ-point, in a lower level structure of ‘topological domains’ linked to single blocks. As similar beads in different blocks can also interact with each other, the long time contact matrices have a more complex, chessboard-like pattern (Fig. 3a), corresponding to a hierarchical organisation of higher-order structures deriving from intra- and inter-domain interactions. By those principles, the combinatorial action of a few different types of binders and corresponding binding sites can produce and regulate an exponential number of 3D conformations and patterns, as we discuss below. While the molecular nature of the blocks envisaged within our SBS framework is yet to be identified in general, a recent study in Drosophila has shown that the blocks could be also linked to local epigenetic patterns^22^. In our view, chromosomal structures discovered in Hi-C data, such as TADs^6^,^7^ and metaTADs^10^, and their differential re-wiring across tissues and cell types, emerge naturally by specialization of the involved molecular factors under general mechanisms of polymer physics.

We also explored some additional, possibly functional consequences of the self-assembly of domains. As TAD boundaries have been associated to biological markers and, more specifically, to an insulating role, we focused on how they affect the physical distance of pairs of sites differently positioned relative to them. Within our toy block-copolymer model, we focused on pairs of sites with the same contour separation: we considered two cases where the pair is located symmetrically or asymmetrically with respect to a domain boundary (Fig. 3b). Interestingly, we found that the block boundary can have a simple symmetry-breaking effect: in the closed phases the sites of the symmetrically positioned pair have a larger physical distance than the asymmetrical pair (p-value = 0), whereas in the open phase no difference is recorded.

**Figure 4:**
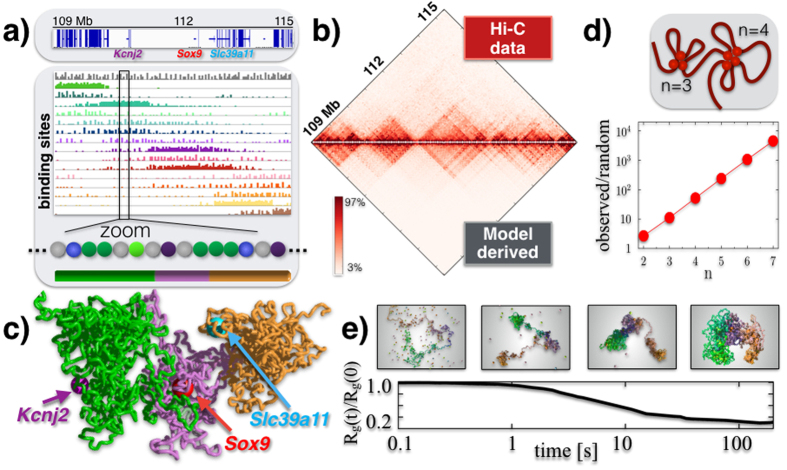
Polymer physics captures the folding of the *Sox9* locus. (**a**) Top: the considered *Sox9* locus in mESC-J1 cells, with a few marker genes. Bottom: the SBS polymer model that best explain the Hi-C contact map of the *Sox9* region has the shown different types of binding sites, as seen in the zoom (different colors); their abundance is represented as an histogram over the genomic sequence. The bar at the bottom highlights three main regional areas to help 3D visualization. (**b**) The model derived pairwise contact frequency matrix (bottom) has a 95% Pearson correlation with Hi-C experimental data (top). (**c**) A snapshot of the *Sox9* locus in its closed disordered state as derived by the polymer model, with the position of TSSs of some key genes highlighted. (**d**) In the locus, many-body contacts of *n* sites are exponentially more abundant than in random SAW conformations (the ratio of the average number is plotted v.s. *n*), which could help the simultaneous co-localization of multiple functional regulatory regions. (**e**) The *Sox9* locus folding dynamics from an initially open conformation towards the closed disordered state is represented by the gyration radius, *R*_*g*_(*t*). Chromatin domains self-assemble hierarchically in higher-order structures, in approx. 20*s*.

Finally, we investigated the occurrence of “many-body” contacts, i.e., co-localization events where multiple sites come simultaneously in physical proximity, beyond pairwise interactions. Within our toy homopolymer model, we measured the frequency of observing *n* sites in physical contact (Figure S5a, see also Fig. 4d and Supplementary Materials and Methods). As expected, in the closed states many-body contacts are exponentially more frequent than in the open state as *n* grows. The contact probability of bead triplets on a same polymer at different genomic separations, *P*_*c*_(*s*_*1*_*, s*_*2*_), is given in Figure S5b. Multiple interactions are currently not detected by Hi-C methods, yet our model highlights that they are likely to be an abundant structural component of chromatin. That hints towards an overseen functional role of closed chromatin domains whereby multiple regulatory regions (enhancers) can loop simultaneously onto a given target (gene promoter) with a much higher probability than in open regions. Taken together our results support a view whereby basic mechanism of polymer folding could play key functional roles in the regulation of the genome by controlling the spatial organisation of chromatin.

### Folding of the *Sox9* locus in mESC

Next, we asked whether our polymer models can also explain the details of folding of specific DNA regions. First, we considered a 6 Mbs sequence around the *Sox9* gene (chr11:109000000-115000000, mm9), an important genomic locus linked to severe congenital diseases^11^, including gene rich areas as well as gene deserts (Fig. 4a). To capture the details of *Sox9*, we generalized our polymer model to accommodate different types of binding sites (colors) and molecular binders (Fig. 4a), and employed a chain with N = 2250 beads (σ = 26 nm) to increase resolution. With a Monte Carlo recursive optimization method (Supplementary Materials), we estimated the minimal arrangement and different types of binding sites that best reproduces the 40 kb Hi-C map available in mESC-J1 cells^6^. We informed our polymer model with the obtained arrangement of binding sites and run full-scale Molecular Dynamics simulations to derive the ensemble of 3D conformations of the locus (see Supplementary Materials and Methods). Figure 4a represents the histograms of the abundance of our inferred binding sites along the locus (different types in different colors), ordered left-to-right according to the location of the domain center of mass. Binding domains tend to overlap with the different TADs in the locus, but importantly they also overlap with each other producing higher-order interactions across TADs, i.e., metaTADs^10^, as visible in the original Hi-C data and in our 3D snapshots as well. In mESC-J1 cells, we find that the mixture best describing the locus is made 64% of the open and 36% of the closed disordered state (see Supplementary Materials and Methods).

The derived pairwise contact frequency matrix has a Pearson correlation with Hi-C data of 95% (Fig. 4b), supporting the view that our polymer model captures a relevant component of the molecular mechanisms determining the folding of *Sox9*. A snapshot of a single typical configuration, in the closed state, is shown in Fig. 4c, where the relative positioning of *Sox9* and other genes in the locus, across its different higher-order domains, can be visualized. For instance, the transcription starting sites (TSSs) of the *Sox9* and *Kcnj2* genes have a genomic separation almost four times larger than the TSSs of *Sox9* and *Slc39a11* (1.72 Mb v.s. 0.46 Mb), but the average physical distances of the two pairs are proportionally closer (1.19 μm v.s. 0.59 μm) as the three genes belong to consecutive regional areas.

The *Sox9* locus is marked by many-body contacts that are exponentially more abundant than expected in a randomly folded conformation (Fig. 4d, error bars within symbol size). The self-assembly of the locus architecture from initial open states proceeds hierarchically, with early formed local domains folding into larger and larger 3D structures encompassing the entire locus (Fig. 4e); the order of magnitude of the time-scale of the process is 20 sec. The variety of information on *Sox9* and its folding mechanisms that can be inferred from polymer physics extends well beyond the Hi-C pair-wise contact data used to infer the model.

To check the general validity of our approach, we applied our polymer models to a different locus, a 2 Mb wide region around the *Bmp7* gene (chr2:171090000-173430000) important in tissue development, where Hi-C data at 30 kb resolution are available in mESC-46C cells^10^. Also in the *Bmp7* case, the inferred SBS binding domains produce a contact matrix having a pattern very similar to experimental data, with a 95% correlation (Figure S6).

### Testing model predictions on the Δ*XTX* deletion in the *Xist* locus

**Figure 5:**
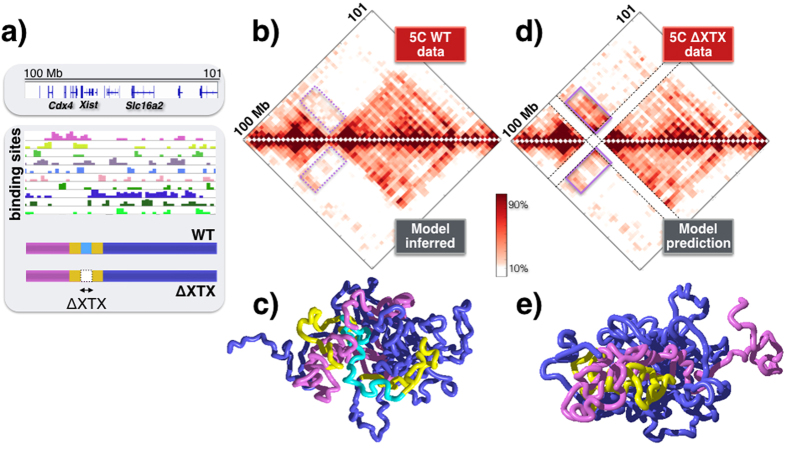
Model predicted effects of the Δ*XTX* deletion in the *Xist* locus. (**a**) The different binding domains are shown of the SBS polymer model of the *Xist* locus, inferred from 5C contact data^7^ in mESC-E14 cells. To help 3D visualization, the bars at the bottom highlight in cyan the wild-type region that is deleted in Δ*XTX* cells, its flanking sequences in yellow and the remaining regional areas overlapping the locus two TADs in violet and blue. (**b**) The model inferred contact matrix (bottom) has a 96% Pearson correlation with 5C experimental data (top). (**c**) A snapshot of the *Xist* locus in its closed disordered state. (**d**) The contact matrix predicted, under no adjustable parameters, by the very same model after the Δ*XTX* deletion (bottom) has a pattern of ectopic interactions (full line magenta box) w.r.t. wild-type data (panel b) very similar to the one seen in the corresponding experimental 5C data (top, correlation 91%). (**e**) Ectopic interactions extend across the yellow regions flanking the Δ*XTX* deletion, as visible in the snapshot of the deleted locus (closed disordered state).

Finally, to test our model, we considered the *Xist* locus, an important 1.3 Mb long region around the *Xist* gene (chrX:100298000-101373000), because data are also available for its Δ*XTX* deletion^7^. Starting from a 20 kb resolution 5C map in mESC-E14 cells^7^, we derived our polymer model describing the wild-type locus and after MD simulations we found a good agreement with 5C data (Fig. 5a,b; a 72% open and 28% closed disordered state mixture returns a 96% Pearson correlation of contact matrices). A 3D snapshot of the locus conformation and its two TADs is shown in Fig. 5c. Next, we implemented the Δ*XTX* deletion in our polymer model and calculated the contact map of the mutated system just from polymer physics, under no fitting or adjustable parameters. We find that the predicted interaction matrix has a pattern of extended ectopic interactions with respect to the wild-type case that is very similar to the one reported in 5C data on Δ*XTX*^7^, with an overall correlation of 91% (Fig. 5d). In the above calculation there are no adjustable parameters. In case the mixture composition is allowed to vary the best fit returns a similar mixture (80–20%), having a 91.4% correlation with the data. Such findings are in agreement with those of a similar interacting polymer model introduced for the *Xist* locus^28^. Our MD simulations show that, after the deletion, the regions flanking Δ*XTX* are spatially repositioned with respect to each other (3D snapshot in Fig. 5e) and contribute to establishing the observed ectopic interactions across regions sharing cognate binding sites. Summarizing, the binding domains identified *in-silico* by our model can describe the folding of *Sox9* with high accuracy and predict the effects of genomic rearrangements based only on polymer physics.

## Discussion

We have discussed a polymer model of chromatin where 3D conformations are shaped by the interactions of binding domains with their cognate binding molecular factors, such as DNA-binding molecules. Genome-wide, and loci specific chromatin contact data can be explained by classical scaling concepts of polymer physics over orders of magnitude in genomic separation, up to chromosomal scales, across mammalian cell types. Consistent with our SBS picture, a model has been recently proposed focused, in particular, on the role of CTFC binding sites and cohesin mediated interactions to explain folding of loci at short scales, in the 10–500 kb range^23^,^29^. In facts, CTFC and cohesin molecules are examples of specific factors and DNA binders that contribute to chromatin folding. Contrary to previous approaches, our model does not require a previous knowledge of the molecular factors responsible for folding (e.g., location of CTCF sites or epigenetics features); conversely, it can be used to infer the nature of the key folding factors.

Pairwise contact data on the *Sox9*, *Bmp7* and *Xist* loci are reproduced by the SBS model with an accuracy above 95%. Additionally, we found that the model predictions on the ectopic interactions induced by the Δ*XTX* deletion in the *Xist* locus are in a remarkably good agreement with available 5C data^7^,^28^. That supports the view that our model captures some of the key folding mechanisms of chromatin. Although current Hi-C technologies only provide information on pairwise contacts, we showed that ‘many-body’ interactions are an abundant, structural component of chromatin 3D organisation.

By combining polymer models and Hi-C data, a quantitative scenario emerges of the large-scale features of chromatin architecture where chromatin folding is determined by a complex system of binding domains and molecular factors, regulated by general mechanisms of polymer physics. As our polymer physics approach identifies the molecular determinants of folding and their mechanisms of action, it can help understanding the link between architecture and function, and the design of novel approaches to personalized diagnosis and treatment of human diseases.

## Materials and Methods

A detailed description of the materials and methods is provided in the Supplementary Materials. In particular, the SBS polymer model was investigated by Brownian Molecular Dynamics computer simulations implemented by LAMMPS. All the details about the model and computational parameters, as well as on the analyses performed are reported in the Supplementary Materials and Methods.

## Additional Information

**How to cite this article**: Chiariello, A. M. *et al*. Polymer physics of chromosome large-scale 3D organisation. *Sci. Rep.* **6**, 29775; doi: 10.1038/srep29775 (2016).

## Supplementary Material

Supplementary Information

Supplementary Figures
