# Supplementary material for: Polymer physics of chromosome large-scale 3D organisation
Source: Sci Rep. 2016 Jul 13;6:29775. doi: 10.1038/srep29775 (PMC4942835; doi:10.1038/srep29775)
Supplement: Supplementary Information [file srep29775-s1.pdf]

Supplementary Materials and Methods

for

Polymer physics of chromosome large-scale 3D organization

Andrea M. Chiariello[1,*], Carlo Annunziatella[1,*], Simona Bianco[1,*], Andrea Esposito[1] and Mario Nicodemi[1,§]

Addresses:

[1]Dipartimento di Fisica, Università di Napoli *Federico II*, and INFN Napoli, CNR-SPIN, Complesso Universitario di Monte Sant'Angelo, 80126 Naples, Italy.

*Equal contribution.

[§] corresponding author:
Mario Nicodemi: mario.nicodemi@na.infn.it

The SBS Polymer Model

In the *String & Binders Switch* (SBS) model[1,2] a chromatin filament is represented as a self-avoiding (SAW) polymer chain, composed by N beads. Each bead of the chain interacts with diffusing Brownian particles (binders) having a molar concentration *c*. The scale of their binding energy to a cognate polymer bead is named E_{int}. The beads of the chain and the binders are subject to Brownian motion, so each particle of the system (bead or binder) obeys the Langevin equation[3]. The friction coefficient of a particle, ζ, in the Langevin equation is related to the viscosity of the solvent η according to Stokes equation $\zeta = 6\pi\eta R$, where R is the radius of the particle. For sake of simplicity, in our simulations we set the diameter of polymer beads and of binders equal to σ, and take it as the unit of lengths. In our simulations we also set the mass of beads and binders equal, and use it as the reference mass unit, *m*=1. We checked that by changing the ratio of the masses our general results remain unchanged, apart from a shift in the time constants of the dynamics.

We use the detailed particle interaction potential developed in classical studies of polymer physics simulations[4]. The potential energy, *V(**x**)*, of a particle at a position, ***x***, has three components. Between any two consecutive beads along the polymer chain there is a potential modeling a finitely extensible spring, the FENE potential[4]. We set the FENE length constant R_0 equal to 1.6σ and *K*, the strength of the FENE spring, equal to $30k_BT/\sigma^2$ (Ref.[4]). To account for excluded volume effects between any two particles there is also a purely repulsive, shifted Lennard-Jones (LJ) potential[4], the so called Weeks-Chandler-Andersen potential, having σ as the length scale unit and $\varepsilon = k_BT$ as the energy scale unit, in the notation used in Ref.[4].

A bead of the polymer interacts with its cognate binders through a short-ranged, attractive Lennard-Jones potential $V_{int}(r)$[5]:

$$V_{int}(r) = \begin{cases} 4\,\epsilon_{int}\left[\left(\frac{\sigma_{b-b}}{r}\right)^{12} - \left(\frac{\sigma_{b-b}}{r}\right)^{6} - \left(\frac{\sigma_{b-b}}{r_{int}}\right)^{12} + \left(\frac{\sigma_{b-b}}{r_{int}}\right)^{6}\right] & r < r_{int} \\ 0 & otherwise \end{cases}$$

where $\epsilon_{int} = \epsilon_{int}{}^{*}\ \varepsilon$ is the control parameter for the intensity of the polymer-binder interaction, $\epsilon_{int}{}^{*}$ is a dimensionless amplitude, r_{int} is the cut-off distance regulating the interaction range and σ_{b-b} is the sum of the radii of the interacting bead and binder. In our case, we set σ_{b-b}=1σ and r_{int} = 1.3σ. The absolute value, E_{int}, of the minimum of the interaction potential, V_{int}, is taken as the energy scale of the interaction; it is proportional to ϵ_{int} through the relation:

$$E_{int} = \left|4\,\epsilon_{int}\left[\left(\frac{\sigma_{b-b}}{r_{int}}\right)^{6} - \left(\frac{\sigma_{b-b}}{r_{int}}\right)^{12} - \frac{1}{4}\right]\right|$$

Molecular Dynamics simulations and physical units

The Langevin dynamics of the system is investigated by Molecular Dynamics (MD) computer simulations implemented by the LAMMPS code via the Verlet algorithm[6]. To model a chromatin filament, we use the polymer model described in the previous section, made of N beads in a box with periodic boundary conditions. The dimensionless friction coefficient is set to ζ=0.5[4]. The linear size of the simulation box, D, is at least as large as the gyration radius of a SAW polymer having the same number of beads ($D \propto N^{0.588}\sigma$).

Given the genomic length L of the chromatin region to be modeled, the corresponding genomic content per chain bead is $s_0 = L/N$. As discussed below, we use N=1000 beads for modeling our homopolymers and blockcopolymers. To give a sense of the scales involved, we consider a chromosome having a genomic length L=100Mb, so in our homopolymer model each bead of the chain contains $s_0 = L/N$=100Kb. The physical diameter of the bead σ is estimated by imposing that the local chromatin density matches the average nuclear DNA density, i.e., by the relation $\sigma \sim \left({s_0}/{G}\right)^{1/3} D_0$ (Ref.[2]), where D_0 is the nucleus diameter and G is the genome length. As we consider mouse embryonic stem cells (mESCs), as an order of magnitude we take D_0 =3.5μm and G = 6.5Gb. For example, in the above case with L=100Mb, the single bead size is σ=87nm. The molar concentration of binders is calculated using the relation $c = P/VN_A$, where N_A is the Avogadro number, V=D^3 is the simulation box volume and P is the number of binders in the box.

The MD dimensionless parameters are mapped into physical units by the standard MD procedure[3,6]. For instance, the time scale, τ, is fixed by the standard MD relation τ=η(6πσ^3/ε). We considered a range of viscosity values around the estimated order of magnitude of the nucleoplasm viscosity[5], and we checked that our general results do not change apart from a time shift in the dynamics. For instance, if η=0.1P at room temperature (T=300K), in a model with N=1000 beads and L=100Mb the time unit is τ=0.03s. We use a MD integration time step $\Delta t = 0.012$[7], and we let the system

evolve up to 10^9 time steps, to reach stationarity. We perform also ensemble averages, up to $5x10^2$ for each particular choice of system parameters.

In all our simulations the polymer is initially prepared in a random SAW configuration, while the binders are randomly located in the simulation box. To produce an initial random SAW configuration we use the following standard approach[4]: we generate a random walk chain where the distance between two consecutive beads is equal to the average length of an equilibrium SAW chain under the FENE and the repulsive Weeks-Chandler-Andersen potentials described above (i.e., 0.97σ)[4]. Then, to remove overlaps between beads and binders, we let the system equilibrate, for 10^7 timesteps, with a soft potential $V_{soft}(r)$ instead of the hard-core LJ repulsion[4]:

$$V_{soft}(r) = \begin{cases} A\left[1 + \cos\left(\frac{\pi r}{2^{1/6}\sigma}\right)\right] & r < 2^{1/6}\sigma \\ 0 & otherwise \end{cases}$$

where the factor A increases linearly in time. The scaling properties of the polymer are then measured to check that the stationary SAW state is attained. Finally, the chain is simulated under the FENE and the repulsive Weeks-Chandler-Andersen potentials, and its scaling properties checked again.

The different types of polymer chains considered

In our investigations we considered different types of models and polymer chains, to describe different types of genomic regions, from the chromosomal scale to the scale of a single locus.

Homopolymer

We studied first a homopolymer of N=1000 identical beads. As seen above, in case we consider a mammalian chromosome of genomic length L = 100Mb, each polymer bead contains approximately $s_0 = \mathrm{L/N}$=100Kb. In our homopolymer model each polymer bead can interact equally with all the binders of the system. In the other cases we considered (listed below), we extend the model to accommodate different types of binding sites along the chain and their specific cognate binders.

Blockcopolymer

As a first generalization of the above described homopolymer model, we investigated blockcopolymers made of two different bead types, each interacting with a specific type of binder. Visually, in Figure 3 we represent the first bead type in red and the second bead type in green, and so their specific binders are colored.

First we considered the case where each polymer block is 500 beads long and the whole polymer is made of 1000 beads in total. To have a sense of scale, we suppose to model a locus of L=10Mb, with σ=64nm and η =2.5cP. The time unit results to be 0.003s. We simulated such a system at different values of the interaction energy ϵ_{int}, ranging from 0 to ϵ_{int}=12k_BT, and we explored a range of concentrations up to *c*=215n*mol/l* for each binder type. In this model, the red and green blocks of the polymer do not interact with each other, and they can fold in each of the three states identified in the homopolymer model: open, closed disordered and closed ordered (see Figure 1b), according to the value of c and ϵ_{int}. In the equilibrium closed states the polymer folds in

conformations where two different domains are spontaneously formed, one composed of red beads and the other of green ones. This is manifested in their corresponding pairwise contact frequency matrix that exhibits two different interaction domains, one for each of the polymer blocks.

As discussed in the Main Text, under the same general conditions we next considered a blockcopolymer having four domains, two red and two green alternated domains (Figure 3), each formed by 250 beads (1000 beads in total), interacting with red and green binders respectively. In this case, the conformation of the polymer in the equilibrium closed states has a hierarchical structure made of two different large domains (one green and one red), each composed of two homologous lower order domains, as seen in checkerboard pattern of the average contact matrix (Figure 3).

Heteropolymer for the *Sox9, Xist* and *Bmp7* locus models

To model at higher-resolution the spatial organization of the *Sox9* locus in mESCs, we use a chain made of N=2250 beads. The locus considered is 6Mb long, and we employed published Hi-C data from Dixon et al., 2012 at 40Kb resolution, from mESC-J1 cells. In this case, the elementary bead in our polymer chain contains about 2.67Kb. The bead size, obtained with the above relation, is σ=26nm. We generalize the models described above and consider a heteropolymer with different types of binding sites and specific cognate binders. The bead types and their positions along the polymer are obtained by a Monte Carlo procedure that minimizes the distance between the Hi-C experimental contact matrix and the simulated contact matrix (see below). In the case of our *Sox9* locus, such a procedure returns 15 different interacting bead types (visually represented by different colors in Figure 4a). Each type of beads of the chain interacts only with its specific type of binders. The interaction between a binder and its cognate bead is modeled by an attractive LJ potential as before, using the following parameters: σ_{b-b}=1σ, r_{int} =1.5σ. Assuming as a reference a viscosity of 2.5cP, the time unit is 0.0002s. In our simulations we sampled values of the total binder concentration, c, ranging from zero to 215n*mol/l*, and we varied the interaction energy from zero

(open state) to ϵ_{int}=16k$_B$T. The concentration and the interaction energy employed for the results discussed in Figure 4 are c=194nmol/l and ϵ_{int}=12k$_B$T, corresponding to a closed disordered polymer state.

Analogously, in the case of the *Xist* locus we use a chain made of N=540 beads. The locus considered is 1.3Mb long and we employed published 5C data from Nora et al. 2012[9] in male undifferentiated WT mESCs (mESC-E14 cells). The fragment based 5C interaction maps have been included in 20kb resolution interaction maps using the online tools my5C[10] (http://my5c.umassmed.edu). Here our procedure returns 10 different interacting bead types. In the case of the deletion ΔXTX[9], we also used 5C data published by Nora et al. 2012 from the XO mES cell line. In the *Bmp7* case we considered a 2Mb wide region around the *Bmp7* gene (chr2:171090000-173430000), where Hi-C data at 30kb resolution are available in mESC-46C cells from Fraser et al. 2015, modelled here by a polymer chain made of N=858 beads. In this case 11 different interacting bead types are found.

We consider an ensemble of up to 5x10^2 independent polymers, each starting from a SAW configuration and equilibrated as described above. Analogously, the binders are initially placed in random positions in the simulation box. To reach equilibrium, we run the simulations up to 10^9 MD time steps.

Method to estimate the organization of the binding sites of our polymer models

To identify the binding domains of the models of the studied loci, we employ a Simulated Annealing Monte Carlo procedure to locate the minimal arrangement of binding sites and types (colors) that, based only on polymer physics, best explains the experimental contact matrix. Our method employs a standard Simulated Annealing scheme and uses a cost function that includes the distance between the input Hi-C and the model predicted contact matrix, and a Bayesian term (a

chemical potential) to penalize overfitting. The details of the procedure will be presented in a more technical publication, Ref.8, devoted specifically to illustrate all the technical aspects of our method.

Order parameters of the phase diagram

The transition lines in the phase diagram of Figure 1b are identified as follows. The coil-globule transition is found by measuring, at equilibrium, the collapse of the gyration radius, R_g, of the polymer[1,2]. R_g has the predicted value of a SAW model when the polymer is in the open state, while it jumps to a much lower value in the compact states (Figure S1a). R_g is defined by the relation: ${R_g}^2 = \frac{1}{M} \sum_{i=1}{}^{N} m_i(r_i - r_{CM})^2$, where m_i and r_i are the mass and the position of the i-th polymer bead, M and r_{CM} are respectively the total mass and the position of the center of mass of the polymer.

The binder order-disorder transition is captured by two structural quantities associated to the spatial configurations of the binders bound to the polymer: their pair distribution function *g(r)* and the structure factor $S(k)$ (Figure S1b), that is related to *g(r)* by definition[3]:

$$S(k) = 1 + 4\pi\rho \int_0^\infty r^2 \sin(kr)/(kr)\, g(r) dr$$

where $\rho = N_b/V$ is the concentration of the binders bound to the polymer. The structure factor $S(k)$ is almost flat in the disordered binder state, while it has sharp peaks in the binder ordered state. The transition order parameter is the ratio $S(k^*)/S_{MAX}$ where k^* is the position of the second peak in *S(k)* and S_{MAX} is a normalization coefficient taken to be equal to the maximum value of $S(k^*)$ across the different considered cases (Figure S2b). The ratio $S(k^*)/S_{MAX}$ has a jump at the order-disorder transition[3]. Analogous results are found in case other peaks of *S(k)* are considered, but the signal to noise ratio can be higher.

Pairwise contact frequency matrices, many-body interactions and fit of Hi-C contact data

The polymer average pairwise contact frequency matrices are obtained in the following way. We fix a contact threshold distance λσ, where σ is the length unit, and λ a dimensionless constant threshold, which we set to λ=3.5. For a given 3D conformation of the polymer chain, we consider the distance r_{ij} between each bead pair i and j, (i≠j, where i,j are bead indices along the chain). If $r_{ij} < \lambda\sigma$, then we count a contact between the beads i and j. We then compute the average of these matrices across the different configurations in the considered polymer state.

The mean contact probability, $P_c(s)$, of a pair of polymer beads having a contour separation, *s* (genomic distance) is recorded in an analogous way by averaging also over all the bead pairs with the same, given contour distance.

To estimate the average number of many-body contacts involving simultaneous interactions of *k* beads occurring in a given polymer conformation, we count the number of beads n_i that are in contact with the *i*-th bead within the above fixed threshold, and the number of possible combinations of *k* simultaneous contacts that contain the *i*-th bead, $\binom{n_i}{k-1}$. We average that number over all the beads in the polymer. As normalization factor, we consider the number of total possible many-body contacts of *k* particles with the *i*-th bead, $\binom{\mathrm{N}}{k-1}$.

The fit of genome-wide Hi-C average pair contact data as a function of the pair genomic separation (Figure 2) is done by use of the Least Square Method (LSM). We compute the model predicted contact probability of a mixture of open and closed states by using the independently derived corresponding contact probabilities from the MD simulations of the homopolymer chain (Figure

1d). Then, by LSM we find the composition of the mixture of open and closed states that minimize the distance between the predicted *P(s)* and the one derived from Hi-C data.

In the case of *Sox9* polymer model, the above procedure is applied to a mixture of the contact matrices of the different states in order to maximize the Pearson correlation coefficient between the model predicted and Hi-C pairwise contact frequency matrices. In this way a Pearson correlation coefficient r=0.93 is obtained. As the resolution is much higher in the *Sox9* case than in chromosome wide simulations, to check the robustness of our approach we also considered a higher threshold value λ=10 and a variant of the procedure where only contacts between monomers of the same color are retained in the calculation of the contact matrix, finding a Pearson correlation coefficient r=0.95 between model predicted and Hi-C data. The data shown in Figure 4b,d are represented in a linear scale (the values corresponding to the 3rd and 97th percentile are highlighted as a reference). The data on the *Xist* locus in Fig.5 and on the *Bmp7* locus in Fig.S6 are analogously treated.

Distance distributions

For measuring the relative physical distances of pairs of sites in the block copolymer model, we focused on two sites A and B, belonging to different consecutive blocks (Figure 3b). We considered two cases where they are located symmetrically or asymmetrically with respect to the block boundary. Importantly, however, in the two cases the contour distance between A and B is kept equal, and set to d=125σ. In the symmetric case, A and B are equally distant from the boundary, whereas in the asymmetric case, one of the two site is placed at a distance 5σ from the boundary. We checked the robustness of our results by considering also larger distances from the domain boundary (e.g., 25σ) and similar results are found.

In the model of the *Sox9* locus, to measure the relative physical distances of the considered genes (*Kncj2*, *Sox9* and *Slc39a11*) we take the genomic coordinates of their TSSs.

Supplementary references

1. Nicodemi, M. & Prisco, A., Thermodynamic pathways to genome spatial organization in the cell nucleus. *Biophys. J.* **96**, 2168-2177 (2009).
2. Barbieri, M., et al., Complexity of chromatin folding is captured by the Strings & Binders Switch model. *Proc. Natl. Acad. Sci. U S A* **109**, 16173-1678 (2012).
3. Allen ,M.P. & Tildesley, D.J., Computer simulation of liquids (Oxford University Press, 1987).
4. Kremer, K. & Grest, G.S., Dynamics of entangled linear polymer melts: A molecular-dynamics simulation. *J. Chern. Phys.* **92**, 5057 (1990)
5. Brackley, C.A. et al., Nonspecific bridging-induced attraction drives clustering of DNA-binding proteins and genome organization. *Proc Natl Acad Sci U.S.A.* **110**, E3605-11 (2013).
6. Plimpton, S. , Fast parallel algorithms for short-range molecular dynamics. *J Comput Phys* **117**, 1–19 (1995).
7. Rosa, A., & Everaers, R., Structure and dynamics of interphase chromosomes. *PLoS Comp. Biol.*, **4**:e1000153 (2008).
8. Bianco, S., et al. in preparation (2016).
9. Nora, E.P., et al., Spatial partitioning of the regulatory landscape of the X-inactivation centre. *Nature* **485**, 381-5 (2012).
10. Lajole, B.R., van Berkum, N.L., Sanyal, A., & Dekker, J., My5C: web tools for chromosome conformation capture studies. *Nature Methods* **6**, 690-1 (2009).
