# Supplementary material for: Polymer physics of chromosome large-scale 3D organisation
Source: Sci Rep. 2016 Jul 13;6:29775. doi: 10.1038/srep29775 (PMC4942835; doi:10.1038/srep29775)
Supplement: Supplementary Figures [file srep29775-s2.pdf]

Supplementary Figures

for

Polymer physics of chromosome large-scale 3D organization

Andrea M. Chiariello[1,*], Carlo Annunziatella[1,*], Simona Bianco[1,*], Andrea Esposito[1] and Mario Nicodemi[1,§]

Addresses:

[1]Dipartimento di Fisica, Università di Napoli *Federico II*, and INFN Napoli, CNR-SPIN, Complesso Universitario di Monte Sant'Angelo, 80126 Naples, Italy.

*Equal contribution.

§ corresponding author:
Mario Nicodemi: mario.nicodemi@na.infn.it

Figure S1

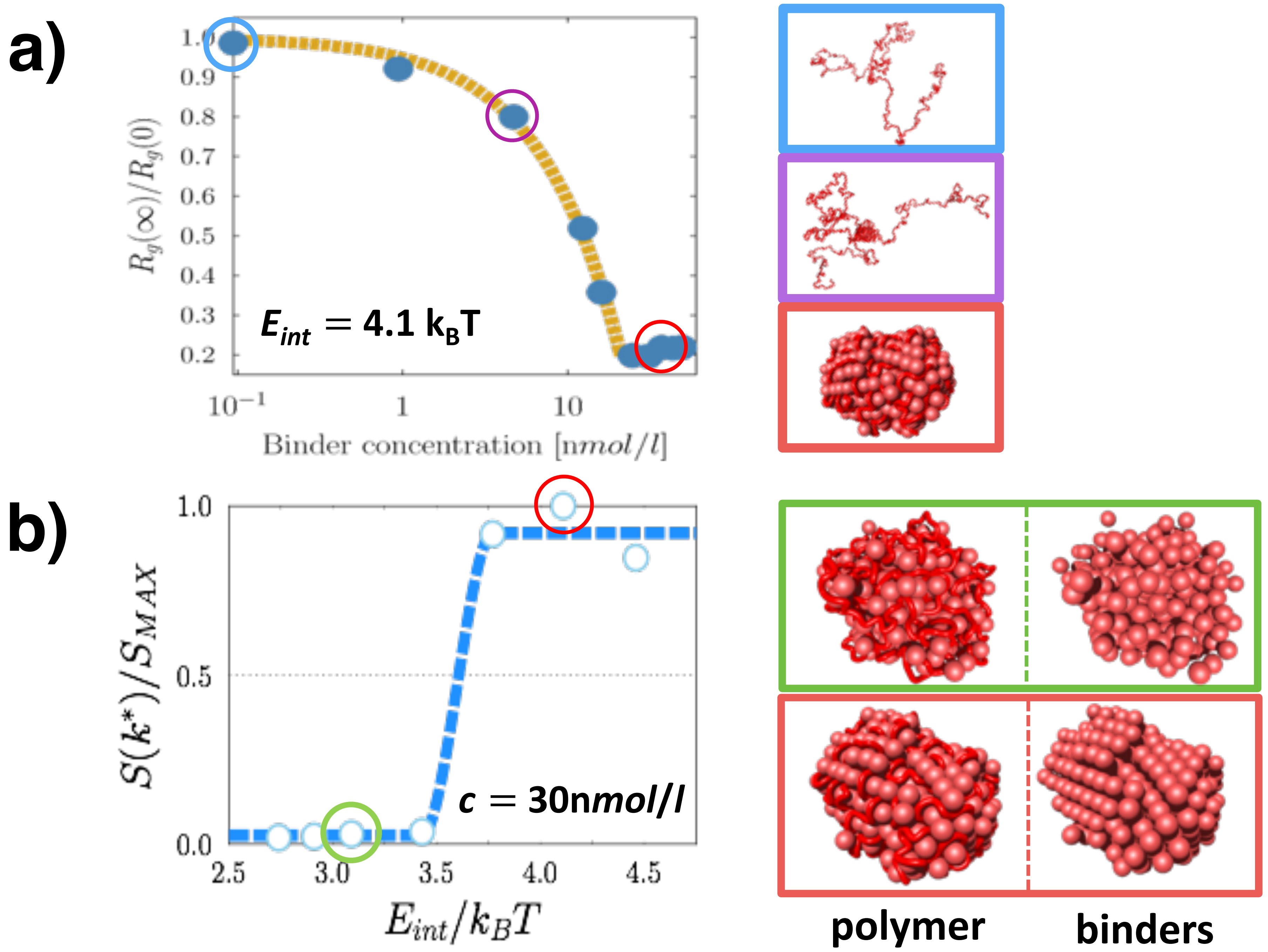

Figure S1

a) The gyration radius of the SBS polymer, R_g, signals its coil-globule transition point as a function of the concentration of binders.

b) The increase of the Structure Factor peak marks the order-disorder transition in the arrangement of the binders around the folded polymer.

Figure S2

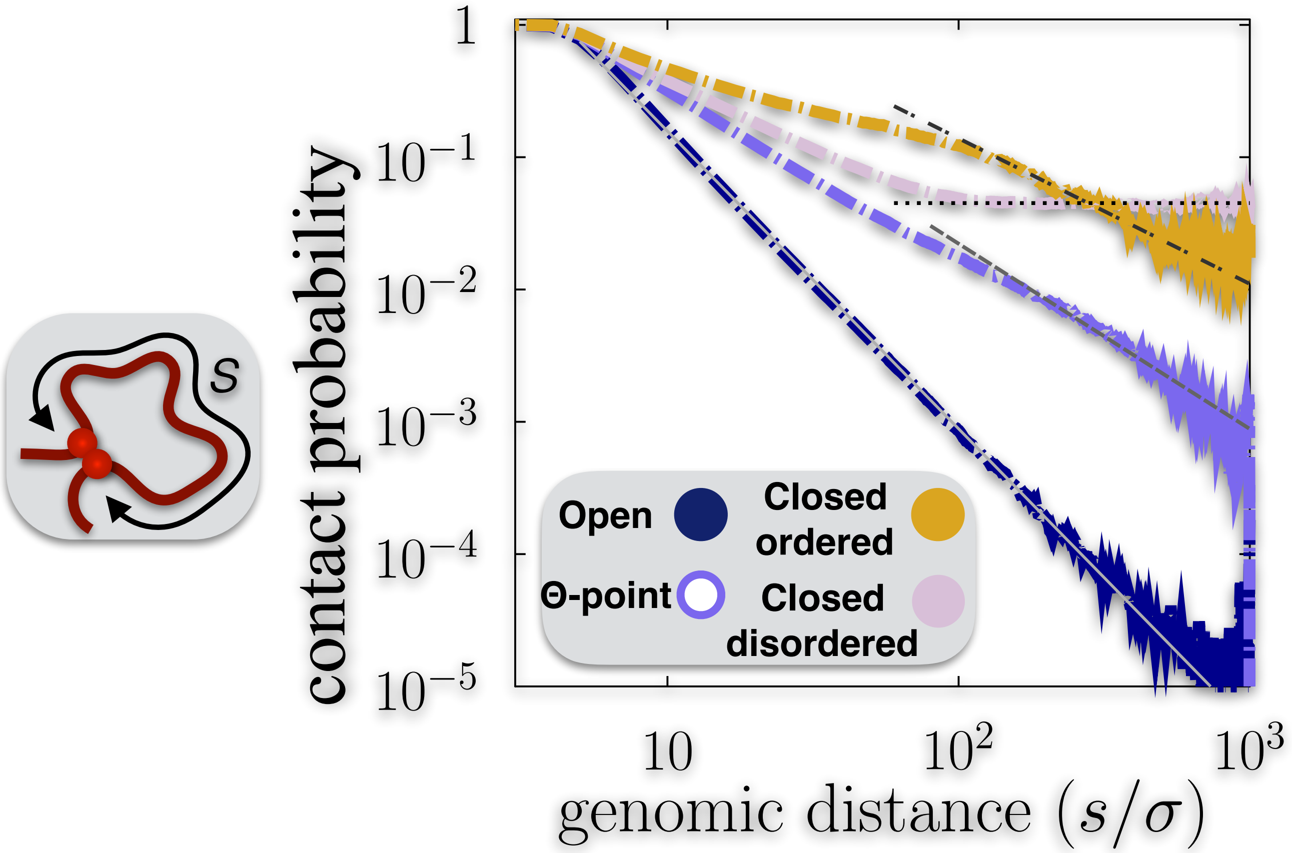

Figure S2
The system pairwise average contact probability, $P_c(s)$, v.s. the contour distance, *s*, in the different phases (see colour circles in Fig.1b)**.** Superimposed are power law fits with exponents predicted by polymer physics (see text).

Figure S3

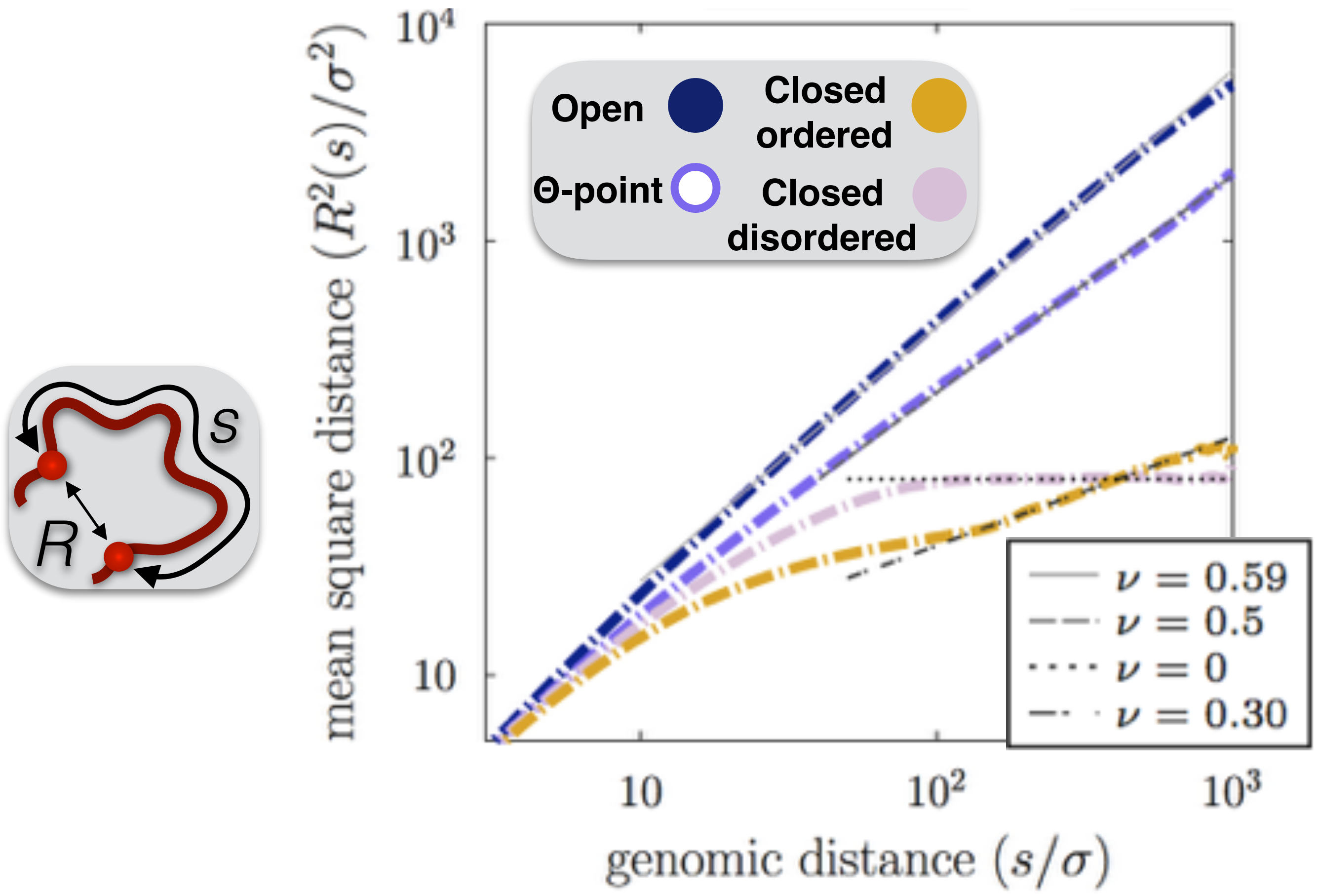

Figure S3

The plot shows the mean square distance, R^2, of two beads having a contour separation s along our SBS homopolymer, in the different thermodynamics phases and at the coil-globule transition point. The asymptotic behaviour is power law, with exponents consistent with the known results from classical polymer physics.

Figure S4

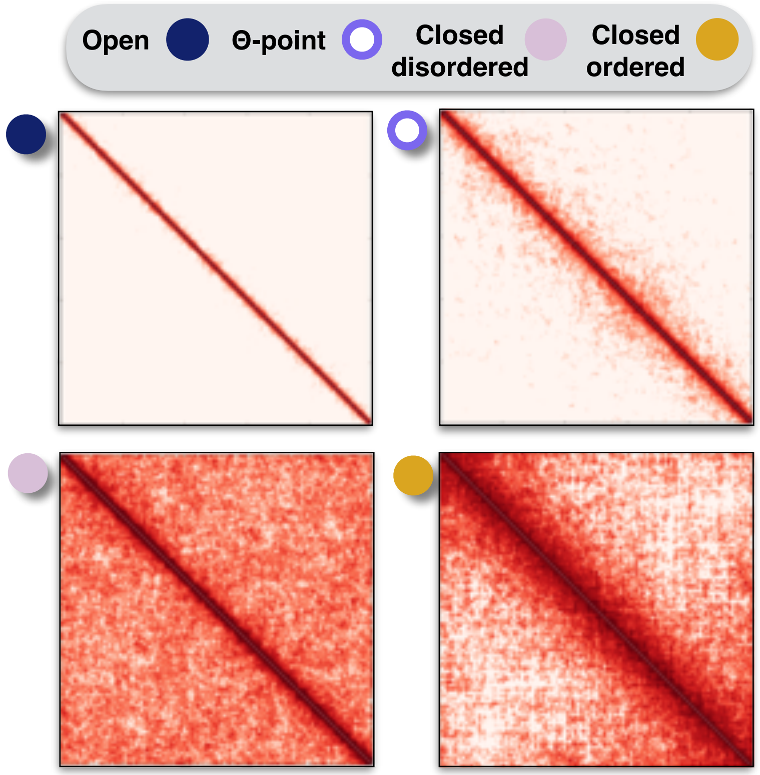

Figure S4
The contact matrices of an SBS homopolymer, made of only one type of binding sites and binders as in Figure 1a, in its stable conformational classes (see colour circles in Fig.1b).

Figure S5

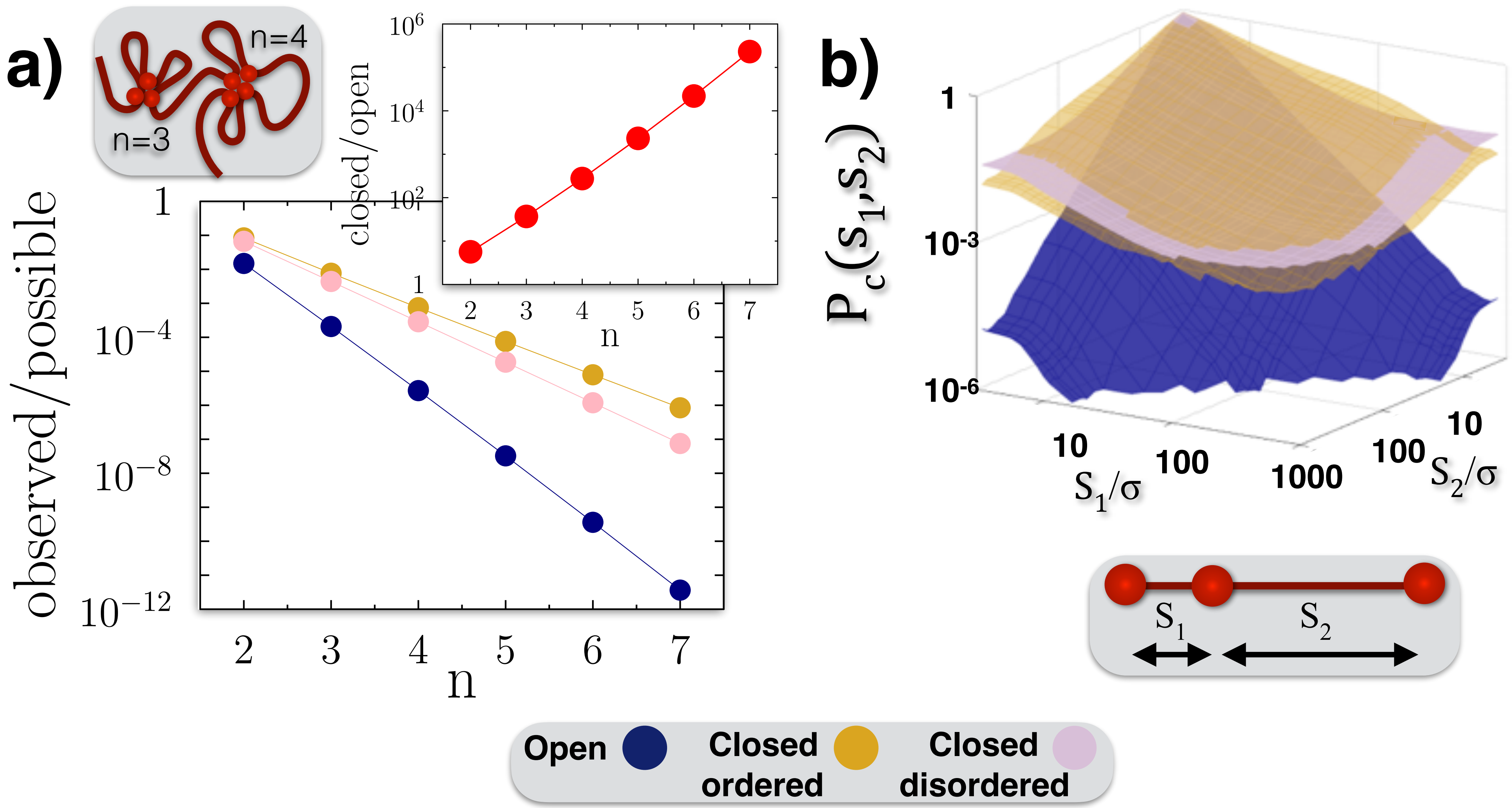

Figure S5

a) The plot shows the frequency, *f(n)*, of observing *n* sites in simultaneous physical contact (normalised by the number of possible combinations of *n* sites) along the SBS homopolymer discussed in the Main Text. The top-left inset shows the ratio of *f(n)* in the compact-disordered and open states.

b) The plot shows the contact probability of bead triplets at different contour separations, $P_c(s_1,s_2)$, along the SBS homopolymer in its different thermodynamics phases.

Figure S6

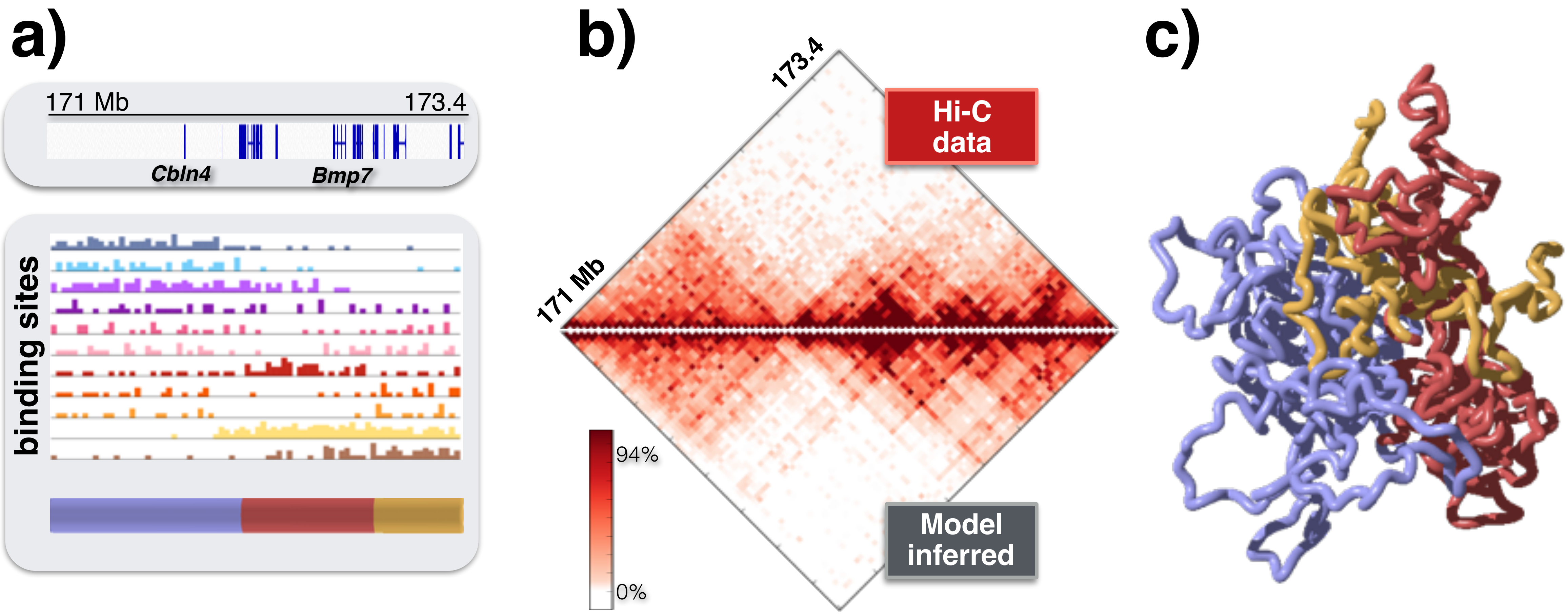

Figure S6.
a) The panel shows the different binding domains of the SBS polymer model of the investigated *Bmp7* locus (chr2:171090000-173430000), as inferred from Hi-C data at 30kb resolution in mESC-46C cells from Fraser et al. 2015. For 3D visualization, the bar at the bottom highlights the regional areas overlapping the locus TADs.
b) The model inferred contact matrix (bottom) has a 95% Pearson correlation with 5C experimental data (top). It corresponds to a 66% open state and 34% disordered globular state mixture.
c) A snapshot of the locus in its closed disordered state.
